# Application research of sensory control management in operating room based on failure mode and effect analysis

**DOI:** 10.3389/fpubh.2026.1826841

**Published:** 2026-07-31

**Authors:** Haijing Cai, Yuehong Ren, Man Xu, Feng Yan, Jiatang Guo, Xinying Liu, Xiangyun Liu

**Affiliations:** 1 Department of Anesthesiology, The 82nd Group Army Hospital of the People's Liberation Army, Baoding, China; 2 Infection Control Office, Baoding Maternal and Child Health Hospital, Baoding, China; 3 Gynecology Department, Baoding Maternal and Child Health Hospital, Baoding, China

**Keywords:** continuous quality improvement, dynamic early warning, failure mode and effect analysis, hospital infection management in operating rooms, risk priority number

## Abstract

**Objective:**

To explore the application effect of Failure Mode and Effects Analysis (FMEA) combined with Risk Priority Number (RPN) dynamic early warning in reducing the risk of hospital-acquired infections in the operating room, and to provide evidence-based support for optimizing infection control management strategies in the operating room.

**Methods:**

This study adopted an analytical and descriptive comparative research design (a prospective pre-post design) and was conducted in the clean operating room of a tertiary hospital from July to December 2023. A FMEA management team consisting of 10 members, including dedicated infection control personnel and members of the operating room infection control group, was formed. Risk identification was carried out using the brainstorming method from the aspects of personnel, equipment, material, method, environment, and monitor. A three-level scoring system (1–10 points) for the occurrence probability (O), severity (S), and detectability (D) of risks was developed based on literature review and clinical practice. The RPN score (RPN = O × S × D) was calculated monthly, and high-risk items were defined as those above the 80th percentile. Targeted interventions were implemented, and the infection control process in the operating room was continuously optimized to improve the quality of infection control management.

**Results:**

A total of 49 risk points were identified, among which 10 were high-risk items. After the intervention, the RPN score of high-risk items decreased significantly, with an improvement rate ranging from 42.86 to 78.57%. The implementation rate of room cleaning and disinfection increased from 51.0 to 85.0% (*P* < 0.05), the correct rate of surgical hand disinfection increased from 66.7 to 90.7% (*P* < 0.05), the compliance rate of hand hygiene increased from 56.7 to 73.6% (*P* < 0.05), the standardization rate of skin disinfection in the surgical area increased from 62.5 to 90.6% (*P* < 0.05), and the standardization rate of skin preparation increased from 71.4 to 95.3% (*P* < 0.05). The pass rate of pre-treatment of surgical instruments increased from 70.8 to 87.5%, but the difference was not statistically significant (*P* > 0.05).

**Conclusion:**

The combination of FMEA and RPN dynamic early warning can effectively identify high-risk links of infection in the operating room. Through and improve the quality of infection control management. This study si a single-center pre-post comparison design. It is recommended that future multi-center randomized controlled trials be conducted to verify its long-term effect.

## 1 Introduction

The operating room is the core place for various surgical operations in a hospital and also a key area for hospital infection control. While patients receive treatment here, they are also exposed to a relatively high risk of hospital-acquired infections (1). According to domestic and international literature reports, the incidence of Surgical Site Infection (SSI) is approximately 2 to 5%, accounting for 14 to 16% of hospital-acquired infections. It not only prolongs the hospital stay of patients and increases medical expenses but also may lead to patient death (2). With the rapid development of surgical techniques and the increasing complexity of surgeries, the traditional infection control model has problems such as lagging and passivity when dealing with infection risks in the operating room, and there is an urgent need to introduce more scientific and dynamic risk management tools.

Failure Mode and Effects Analysis (FMEA) is a systematic and forward-looking risk assessment method that originated from the aviation and automotive industries and has been widely applied in the field of medical quality improvement in recent years (3, 4). FMEA identifies potential failure points, quantifies risk priorities, and formulates targeted intervention measures, enabling prevention before adverse events occur, which is in line with the modern hospital infection management concept of “prevention first.” However, current study on surgical room infection control in China mostly focuses on static indicator monitoring (such as air bacterial colony counts, hand hygiene compliance) (5, 6), but lacks systematic analysis of the risk occurrence mechanism and key links.

In the field of surgical room risk analysis, Zhao et al. (11) applied Failure Mode and Effects Analysis to systematically identify the hospital infection risks in the central surgical room and established a risk assessment system covering multiple dimensions such as personnel, equipment, and environment. Guo et al. (7) constructed a surgical room hospital infection risk assessment tool and achieved risk classification management through quantitative scoring. Xu et al. (15) systematically expounded the implementation and application of hospital infection risk management methods, providing methodological guidance for medical institutions to conduct risk assessment. These studies laid a theoretical foundation for the application of FMEA in surgical room infection control management, but still lacked research on a continuous quality improvement mechanism based on RPN dynamic warning.

## 2 Objectives and scope of the study

This study aims to apply the FMEA method to surgical room infection control management. By constructing a dynamic cycle mechanism of “identification- assessment-intervention-reevaluation,” it realizes early warning and precise control of infection risks (8, 10). The innovation points of this study are: (1) establishing a dynamic early warning system based on RPN score, breaking through the limitations of traditional static monitoring; (2) adopting a multi-dimensional risk assessment framework (personnel, equipment, material, method, environment, and monitor), covering all aspects of surgical room infection risks; (3) verifying the practical effect of FMEA in surgical room infection control management through continuous quality improvement cycles. The study results provide a quantifiable and replicable infection control management improvement path for medical institutions, which has significant practical significance for improving surgical safety.

## 3 Study methodology

### 3.1 Study design

This study adopted an analytical and descriptive comparative research design (a prospective pre-post design) and utilized the FMEA risk management tool. It was conducted following the process of “team formation and training → risk identification and analysis → risk evaluation.” The study was carried out in the clean operating room of a tertiary hospital in Hebei Province from July to December 2023. The reason for choosing this operating room was as follows: (1) As a regional medical center, it has an annual surgical volume of over 11,000 cases, which is representative; (2) The operating room is well-equipped (200-level rooms for 2 surgeries and 1700-level rooms for 17 surgeries), covering various types of surgeries; (3) The hospital's infection management is relatively good, and the conditions for implementing FMEA are available. This study strictly adheres to medical ethical principles and has been reviewed and approved by the Institutional Ethics Committee of Baoding Maternal and Child Health Hospital (Apprpval No.: 2023-01-K0012). The entire study process only involves optimizing the infection management procedures in the operating room and does not collect patients' personal information or affect clinical treatment. All analyzed infection control data have been anonymized. The RPN scores and improvement measures are managed by authorized personnel to ensure information security. The study enhances surgical safety through prospective risk assessment, and only adds necessary training burdens to medical staff without introducing additional risks. There are no conflicts of interest to declare.

### 3.2 Formation and training of the FMEA management team

A surgical room FMEA management team was formed by 5 hospital infection management professionals and 5 members of the surgical room infection control team (including 5 with senior titles, 4 with intermediate titles, and 1 with junior title, with all members having more than 5 years of surgical room work experience). Before the study began, a 2-week standardized training was conducted for the team members, covering: (1) the basic principles and implementation steps of FMEA; (2) interpretation of risk scoring standards and case practice; (3) training on inter-rater reliability. After the training, a consistency test was conducted, and participants were allowed to participate in the formal scoring only if the Kappa coefficient was ≥ 0.75. The final inter-rater reliability Kappa coefficient was 0.766, achieving a good level of consistency (9).

### 3.3 Identification and analysis of hospital infection risks

Members of the FMEA management team used the brainstorming method to identify hospital infection risks in the operating room from the aspects of personnel, equipment, material, method, environment, and monitor (7). The brainstorming meeting was held once a month, each session lasting 2–3 h. The topics discussed included: analysis of typical cases of hospital infection in domestic and international operating rooms and identification of weak links in the current infection control process. Risk is defined as a potential failure link in the diagnosis and treatment activities in the operating room that may cause hospital infection in patients or occupational exposure for medical staff (10). According to the source of risk, it is classified into three categories: systematic risk (defects in systems and processes), operational risk (deviations in personnel behavior), and environmental risk (problems with facilities and equipment), corresponding to different activity links in the operating room.

Based on literature review and combined with actual clinical work, the levels of the three dimensions of possibility (O), severity (S), and detectability (D) of the risks were set and assigned score, and the surgical room hospital infection risk assessment form and assessment criteria were formulated. The basis for the scoring standard formulation: (1) possibility of occurrence refers to the epidemiological data of hospital infection rates in domestic and international operating rooms; (2) severity is based on the impact of infection on the patient's prognosis and the length of hospital stay; (3) detectability is based on the sensitivity and timeliness of existing monitoring methods (11–14) (Tables 1–3).

**Table 1:** Scoring criteria for the severity level of results caused by risks.

Severity level	Scoring criteria	Score
Catastrophic	It can lead to an outbreak of nosocomial infections and cause severe injuries or death to patients	10
Critical	It can lead to SSI in patients, requiring reoperation or an extension of ICU stay of more than 7 days	9
Serious	It is very likely to cause SSI, requiring an extension of the hospital stay by 5 to 7 days and significantly increasing medical expenses	8
Moderately serious	It is very likely to cause SSI, resulting in more serious harm to the patient and prolonging the hospital stay by 3 to 5 days	7
Generally serious	It may lead to SSI, cause general injury to the patient, and may prolong the hospital stay by 1 to 3 days	6
Generally	It can lead to sporadic SSI, with a controllable range, causing minor harm to the patient	5
Moderately mild	It is not likely to cause SSI, but it may cause temporary discomfort or a decrease in patient satisfaction	4
Mild	It is not likely to cause SSI and only causes temporary and mild discomfort to the patient	3
Very mild	It has no clinical impact on the patient and is only recorded as an abnormal event	2
No	No influence	1

**Table 2:** The scoring criteria for the incidence rate of risks.

Probability of occurrence	Probability of possible occurrence	Score
Extremely high	≥1/2 (almost every surgery is likely to occur)	10
Very high	1/3	9
High	1/8 (reported high incidence of SSI)	8
Relatively high	1/20	7
Moderate	1/80	6
Moderately low	1/200 (reported average incidence of SSI)	5
relatively low	1/800	4
Low	1/2,000	3
Very low	1/10,000	2
Extremely low	≤ 1/15,000 (rare event)	1

**Table 3:** Scoring criteria for the detectability level of risks.

Detectability	Evaluation criteria	Score
Almost certain	The current monitoring methods can almost certainly detect (such as daily on-site inspections)	1
Very high	There is a high probability of being detected (such as through real-time electronic monitoring systems)	2
High	There is a high probability of being detected (such as during weekly quality control inspections)	3
Relatively high	There is a relatively high probability of being discovered (such as during monthly quality control spot checks)	4
Moderate	There is a moderate chance of being discovered (such as during quarterly spot checks)	5
Relatively low	There is a low chance of being discovered (such as in the semi-annual review)	6
Low	There is a very low chance of being found to rely on adverse event reporting	7
Very low	There is a very small chance of being discovered (a special investigation is required to identify it)	8
Extremely low	There is a very small chance of being discovered (with extremely strong concealment)	9
Absolutely undetectable	It will not and/or cannot be discovered	10

### 3.4 Risk assessment

The formula for calculating the Risk Priority Number (RPN) is RPN = Probability of Risk Occurrence (O) × Severity of Risk (S) × Detectability of Risk (D). The RPN score for each risk factor ranges from 1 to 1000. Assign a score to each risk factor according to the formula, and the result is the risk score of the risk item. Based on the distribution characteristics of the 49 risk RPN score, it is divided into five levels according to the 20th, 40th, 60th, and 80th percentiles: Low Risk (RPN ≤ P20), Moderate-Low Risk (P20 < RPN ≤ P40), Moderate Risk (P40 < RPN ≤ P60), Moderate-High Risk (P60 < RPN ≤ P80), and High Risk (RPN > P80). In this study, P80 = 144 points. Therefore, RPN > 144 points are defined as high-risk items, and priority intervention is required (15).

### 3.5 Observation indicators

#### 3.5.1 Outcome indicators

(1) Changes and improvement rates of RPN score for high-risk failure modes (Improvement Rate = (Pre-intervention RPN–Post-intervention RPN)/Pre-intervention RPN × 100%); (2) Incidence of surgical site infection (SSI) (Due to the limitation of the study period, the SSI data need to be extended for follow-up. This study mainly reports process indicators).

#### 3.5.2 Process indicators

(1) Implementation rate of interoperative cleaning and disinfection; (2) Correct rate of surgical hand disinfection; (3) Hand hygiene compliance rate; (4) Standardization rate of skin disinfection in the surgical area; (5) Standardization rate of skin preparation; (6) Qualified rate of pre-treatment of surgical instruments. All indicators are evaluated according to industry standards such as “Guidelines for Surgical Room Nursing Practice” and “Standards for Medical Staff Hand Hygiene.”

### 3.6 Statistical analysis

Data analysis was conducted using SPSS 27.0 statistical software. Qualitative data were expressed as frequencies and percentages (%). The χ^2^ test or Fisher's exact test was used for comparison before and after the intervention. The effect size (Phi coefficient) and 95% confidence interval were reported. Two-sided test, with *P* < 0.05 considered statistically significant. Sample size estimation: Based on pre-test data, assuming that the hand hygiene compliance rate increases from 60 to 80% after the intervention, with α = 0.05 (two-sided) and β = 0.20, the required minimum sample size for each group was calculated to be 91 cases. The actual sample size of this study met the requirements.

### 3.7 Risk response and intervention measures

For the high-risk projects identified, the FMEA team employed the Root Cause Analysis (RCA) method. Through fishbone diagrams and 5 why analysis, they traced the causes of failure and formulated intervention strategies based on evidence-based medicine. The intervention measures of the FMEA team were implemented in six dimensions: “personnel, equipment, material, method, environment, and monitor.”

## 4 Results and discussion

### 4.1 Identification results of hospital infection risks in the operating room

Through systematic assessment, a total of 49 risk points were identified from six dimensions: personnel, equipment, material, method, environment, and monitor (Supplementary Table 1). The high-risk items mainly concentrated in the “personnel” (medical staff's lack of aseptic awareness, RPN = 168), “equipment” (failure of air conditioning and purification system, RPN = 175; improper disinfection of anesthesia machine, RPN = 168), “method” (inadequate implementation of operating room regulations and norms, RPN = 180), “environment” (inadequate cleaning of environmental surfaces in the operating room, RPN = 175; unsatisfactory air self-purification during consecutive surgeries, RPN = 168), and “monitor” (low compliance rate of hand hygiene, RPN = 210; incorrect surgical hand disinfection, RPN = 175; non-standard disinfection of the surgical area skin, RPN = 180; failure to monitor the concentration of disinfectant, RPN = 160). No high-risk items were evaluated in the “material.”

### 4.2 Evaluation of intervention effects for high-risk projects

#### 4.2.1 Changes in RPN score of the improvement projects

After 6 months of continuous intervention, the RPN score of 10 high-risk projects all significantly decreased (Table 4), with improvement rates ranging from 42.86 to 78.57%. The projects that showed the most significant improvement included: improper disinfection of anesthesia machine (improvement rate 78.57%, RPN from 168 to 36), failure of air conditioning purification system (improvement rate 76.00%, RPN from 175 to 42), and poor aseptic awareness among medical staff (improvement rate 73.21%, RPN from 168 to 63).

**Table 4:** Changes in RPN score before and after intervention for high-risk projects.

Risk control project	Before intervention (July 2023)				After intervention (December 2023)				Improvement rate (%)	Improve intervention strategies
	S	O	D	RPN	S	O	D	RPN		
Low hand hygiene compliance rate	7	6	5	210	5	6	4	120	42.86	①Develop a tiered training plan and record videos of hand hygiene procedures; ② Conduct on-site inspections and video monitoring for behavior monitoring; ③ Incorporate into performance evaluation.
Inconsistent skin disinfection in the surgical area	6	5	6	180	3	5	4	60	66.67	① Specialized training for junior physicians; ② Video learning on skin disinfection procedures in the surgical area.
Inadequate implementation of surgical room regulations	6	6	5	180	3	6	4	72	60.00	① Revise and improve the management system at both the hospital and department levels; ② Develop aseptic technique operation procedures; ③ Conduct practical teaching through “simulated surgeries.”
Insufficient cleaning and disinfection of environmental surfaces in the operating room	7	5	5	175	4	5	4	80	54.29	① Implement the responsibility management system for visiting nurses; ② Conduct questionnaire surveys for cleaning staff and provide on-site training and assessment; ③ Supervise and implement the tasks by the responsible team leaders.
Incorrect surgical hand disinfection	7	5	5	175	3	5	4	60	65.71	① Specialized training and assessment on surgical hand disinfection; ② Monitoring of the effectiveness of surgical hand disinfection; ③ Key training for junior physicians.
Failure of the air purification system or inadequate maintenance	5	7	5	175	2	7	3	42	76.00	① The maintenance department formulates maintenance procedures and Gantt charts; ② Filters and filter screens are promptly cleaned and replaced; ③ Regular maintenance records are checked.
Air self-purification fails to meet standards between consecutive surgeries	7	6	4	168	3	6	3	54	67.86	① Strictly manage the interval time between consecutive surgeries; ② Monitor the air purification effect; ③ Optimize the surgical scheduling.
Inappropriate disinfection and handling of anesthesia machines	7	6	4	168	2	6	3	36	78.57	① Assign a dedicated person to be responsible for cleaning and disinfecting the internal pipelines of the anesthesia machine; ② Develop standard operating procedures for the maintenance of the anesthesia machine.
Poor aseptic awareness among medical staff	6	7	4	168	3	7	3	63	73.21	① Group-based and tiered training plan; ② On-site inspection combined with video monitoring for behavior monitoring; ③ Incorporation into performance assessment and reward/punishment mechanism.
Concentration of the disinfectant was not monitored as per the requirements.	8	5	4	160	3	5	3	45	71.88	① Use transparent covered containers to store the disinfectant; ② Mark the graduations on the outer surface of the containers; ③ Clearly indicate the mixing ratio; ⑤ Regularly monitor the concentration.

#### 4.2.2 Improvement in process indicator score

Compared between December 2023 (after intervention) and July (before intervention), all infection control process indicators showed significant improvement (Table 5). The implementation rate of interoperative cleaning and disinfection increased from 51 to 85% (χ^2^ = 15.740, *P* < 0.001), the correct rate of surgical hand disinfection increased from 67 to 91% (χ^2^ = 24.000, *P* < 0.001), the hand hygiene compliance rate increased from 57 to 74% (χ^2^ = 4.182, *P* = 0.041), the standardization rate of skin disinfection in the operating area increased from 63 to 91% (χ^2^ = 7.053, *P* = 0.008), and the standardization rate of skin preparation increased from 71 to 95% (χ^2^ = 13.414, *P* < 0.001). The qualified rate of pre-treatment of surgical instruments increased from 71 to 88%, with no statistically significant difference (χ^2^ = 2.021, *P* = 0.155).

**Table 5:** Changes in infection control indicators in the operating room before and after intervention.

Hospital infection indicators	Before intervention (July 2023)	After intervention (December 2023)	*X* ^2^	*P*	Effect size (ϕ)	95% CI
Implementation rate of interoperative cleaning and disinfection	0.51 (29/57)	0.85 (51/60)	15.740	0.000	0.36	0.20–0.52
Correct rate of surgical hand disinfection	0.67 (80/120)	0.91 (136/150)	24.000	0.000	0.30	0.18–0.42
Hand hygiene compliance rate	0.57 (34/60)	0.74 (53/72)	4.182	0.041	0.17	0.01–0.33
Standardization rate of skin disinfection in the operating area	0.63 (20/32)	0.91 (29/32)	7.053	0.008	0.33	0.09–0.57
Standardization rate of skin preparation	0.71 (50/70)	0.95 (61/64)	13.414	0.000	0.30	0.14–0.46
Qualified rate of preoperative treatment of surgical instruments	0.71 (17/24)	0.88 (21/24)	2.021	0.155	–	–

### 4.3 Implementation of intervention strategies based on FMEA

For high-risk projects, the study team formulated and implemented multi-dimensional intervention strategies: (1) Strategies targeting “personnel” factors: Develop training plans for different groups and levels in the operating room, and record videos such as “sterilization of surgical hands,” “hand hygiene,” and “skin disinfection in the surgical area.” Focus training on low-experience physicians on surgical hand disinfection and skin disinfection in the surgical area, and monitor the effect of surgical hand disinfection; conduct training on environmental cleaning and disinfection and cleaning tool handling for cleaning staff; and provide training on surgical hand disinfection, sterile package packaging, and instrument transfer for operating room instrument nurses. For the common situation of older age, low educational level, and lack of protection awareness among cleaning staff, the infection control full-time personnel conducted a questionnaire survey first, then on-site training and assessment, and evaluated the effect of environmental surface cleaning and disinfection. Enhance the infection prevention and control ability of cleaning staff. Monitor the behavior awareness of personnel through on-site inspections and by retrieving surveillance videos of the operating room at different times. Incorporate the monitoring results into the hospital's performance assessment to enhance safety awareness. (2) Strategies targeting “equipment” factors: The maintenance of the air purification system is jointly completed by the power department and the operating room. The power department formulates the maintenance process and Gantt chart for the air purification system to ensure timely cleaning or replacement of filters and filters. A dedicated position is set up for the pre-treatment of surgical instruments, and the laparoscopic instruments are handled by the laparoscopic team members. The nurses in the disinfection supply center and the quality control personnel in the operating room jointly monitor the quality of pre-treatment of surgical instruments and promptly report any problems to the operating room for immediate improvement. Assign a dedicated person to be responsible for the cleaning and disinfection of the internal pipelines of the anesthesia machine. (3) Strategies targeting “material” factors: Use transparent covered containers to store disinfectants, and mark the outer surface of the containers with scale indications, indicating the proportion of chlorine-containing disinfectant. Use different color markings for cleaning ground towels in different areas, and use numbers for small towels that are wiped. Avoid repeated soaking and use of tools. (4) Strategies targeting “method” factors: The hospital infection management system for the operating room and the standards for pre-treatment of surgical instruments and sterile technique operation procedures were revised and improved at the hospital department level. Training and assessment were conducted for instrument nurses and circulating nurses, and “simulation surgery” practical training was provided for new employees. Conducting evaluations and awards for outstanding instructors and students, and formulating reward measures to promote the implementation of the regulations at all levels in the operating room. Conducting competitions for outstanding instructors and students, and formulating reward measures to promote the implementation of regulations at all levels in the operating room. (5) Strategies targeting “environment” factors: Implement the responsibility management system for circulating nurses in the operating room. Arrange circulating nurses according to the surgical level and be fully responsible for the quality of the operating room on the same day. Guide and assist cleaning staff in completing consecutive surgeries and final cleaning and disinfection work. The responsibility group leader is responsible for supervising and implementing the weekly cleaning and disinfection. Members of the infection control team conduct regular monitoring of the quality of environmental cleaning and disinfection through on-site inspections and environmental hygiene monitoring.

### 4.4 Discussion

Hospital infection risk management is the core of hospital infection management work. To further strengthen the prevention and control of infections in medical institutions, the Office of the National Health Commission issued ten basic infection prevention and control regulations in 2019. The “Infection Control Risk Assessment System” became one of the ten core regulations for infection prevention and control. Risk management has been widely applied in hospital infection management work (16). For instance, Wang et al. (17) used risk management to improve the quality of medical device management in the operating room and the satisfaction of surgeons, Ma (18) applied risk management to improve the quality of hospital infection management in laminar flow clean operating rooms, and all the infection prevention and control work achieved significant results.

This study applied the forward-looking risk management tool FMEA to the infection control management of the operating room. By constructing a dynamic cycle mechanism of “identification—assessment—intervention—re-assessment,” it achieved early warning and precise control of infection risks. The study results showed that after 6 months of intervention, the RPN score of 10 high-risk items significantly decreased, with an improvement rate ranging from 42.86 to 78.57%, and various infection control process indicators significantly improved, verifying the effectiveness of FMEA in operating room infection control management. Compared with the research conducted by Zhao et al. (11), this study not only identified the infection risks in the operating room but also established a warning system based on the dynamic threshold of RPN, achieving continuous monitoring of risks and precise intervention. Compared with the static assessment tool constructed by Guo et al. (7), this study broke through the limitations of traditional “one-time assessment” by calculating the RPN score monthly and dynamically adjusting the intervention strategies. Moreover, the improvement rate (42.86%−78.57%) of this study is consistent with the results of Wang et al. (17) who applied risk management to enhance the quality of surgical room medical device management (with an increase in satisfaction of approximately 30%), and Ma's (18) research on improving the infection management quality of laminar flow clean operating rooms. However, this study has more advantages in terms of the precision of risk quantification and the targeted nature of intervention.

Compared with traditional static monitoring, FMEA has the following advantages: Firstly, forward-looking prevention. FMEA identifies potential risks before adverse events occur, which conforms to the modern hospital infection management concept of “prevention first.” Among the 49 risk points identified in this study, 80% are potential risks that have not yet caused actual infection events, demonstrating the predictive score of FMEA. This is consistent with the predictive concept of the medical failure mode algorithm proposed by Kobo-Greenhut et al. (22), which is to identify potential failure links in advance through systematic analysis. Secondly, systematic assessment. Through the six-dimensional framework of “personnel, equipment, material, method, environment, and monitor,” it comprehensively covers the infection risk links in the operating room, avoiding the one-sidedness of traditional monitoring. Thirdly, quantification of priority. The RPN score converts subjective risk perception into objective score, providing a scientific basis for resource allocation and intervention decisions. This study set the 80th percentile as the threshold to ensure that high-risk projects receive priority intervention, which is superior to the traditional empirical risk assessment methods.

This study found that high-risk items mainly concentrated in the “person” and “measurement” dimensions. Hand hygiene compliance (RPN = 210) and surgical hand disinfection (RPN = 175) ranked first and second, which is consistent with the core role of hand hygiene in preventing healthcare-associated infections as reported by Allegranzi and Pittet (19), and also aligns with the importance of behavioral intervention emphasized in the WHO (20) guidelines on core components of infection prevention and control. In this study, the hand hygiene compliance rate increased from 57 to 74%, which is consistent with the improvement trend of behavioral indicators reported by Xu Yan et al. (15) after the implementation of hospital infection risk management methods. The weak awareness of asepsis among medical staff (RPN = 168) is the fundamental reason, which is consistent with the result identified by Zhao et al. (11) as a high-risk factor of “personnel factors,” suggesting that behavioral intervention and cultural construction are the key breakthrough points for infection control management in the operating room. Environmental-related risks (air conditioning purification system, operating room cleaning and disinfection) also deserve attention, as the physical environment of the clean operating room is the basic guarantee for preventing SSI (21).

Notably, the qualified rate of surgical instrument pre-treatment (70.8% vs. 87.5%, *P* = 0.155) did not reach statistical significance. Possible reasons include: (1) The sample size was relatively small (*n* = 24), and the test power was insufficient; (2) The intervention period was short (6 months), and behavioral changes needed more time to be solidified; (3) The pre-treatment of surgical instruments involved multi-department collaboration (operating room—disinfection supply center), and the complexity of process optimization was high. This is similar to the experience of Wang et al. (17) in their study regarding the improvement of medical device management quality. Their study also pointed out that the optimization of the multi-department collaboration process requires a longer intervention period. It is recommended to extend the observation period in the future and use interrupted time series analysis to evaluate the long-term effects. At the same time, referring to the failure mode analysis method based on simulation by Balmaks et al. (12), the pre-treatment process of the devices should be further optimized.

Although this study has achieved certain results, it still has the following limitations: (1) Study design: The single-center pre-post control design cannot completely eliminate confounding factors such as time trends and Hawthorne effect, and the strength of causal inference is limited. It is recommended that future multi-center cluster randomized controlled trials or stepped wedge design studies be conducted. (2) Sample limitations: The study is based solely on data from a single center and does not include multi-center controls, which may affect the generalizability of the results. In the future, the sample size should be expanded to further verify the applicability of the FMEA model in different medical institutions. (3) Insufficient time span: The intervention period is 6 months, and the effects of some long-term measures (such as system revisions) need to be observed over a longer period. (4) Subjective scoring bias: The RPN score relies on the subjective judgment of the group members. Although training was conducted to ensure the reliability among scorers (Kappa = 0.766), there is still potential bias. In the future, objective indicators (such as microbial monitoring data, electronic hand hygiene monitoring system) can be introduced to assist in verification.

Based on the results of this study, future efforts should focus on: (1) the long-term impact of FMEA intervention on the incidence of SSI; (2) comparative studies on the implementation effects of FMEA in different medical institutions; (3) the integrated application of FMEA with other quality management tools (such as PDCA and quality control circles).

## 5 Conclusion

Previous studies have confirmed that the risk assessment RPN score can reflect the consensus of the assessment experts on the severity of risks (22). This study has verified that the FMEA method can effectively identify high-risk links of surgical room infections and achieve precise intervention through dynamic RPN monitoring. Through multi-dimensional risk assessment and cross-departmental collaboration, the infection control system and operation procedures have been optimized, significantly improving the management quality. Although the qualified rate of pre-treatment of surgical instruments has not improved yet, a continuous improvement mechanism has been established. Subsequently, it is necessary to strengthen supervision and supplement the sample size. FMEA provides a systematic and scientific management tool for surgical room infection control, and it is worthy of promotion in medical institutions. However, the implementation plan needs to be adjusted in accordance with local conditions.

## Data Availability

The original contributions presented in the study are included in the article/Supplementary material, further inquiries can be directed to the corresponding author.
