# Supplementary material for: Application research of sensory control management in operating room based on failure mode and effect analysis
Source: Front Public Health. 2026 Jul 31;14:1826841. doi: 10.3389/fpubh.2026.1826841 (PMC13473422; doi:10.3389/fpubh.2026.1826841)
Supplement: Supplementary file 1 [file Table_1.docx]

**Supplementary table 1. The RPN score of risk points in the operating room risk assessment system in July 2023**

| **Risk categorie** | | **Potential failure mode** | **Severity of consequences（S）** | **Possibility of risk occurrence（O）** | - **Detectability（D）** | **Score** | **Risk grade** |
| --- | --- | --- | --- | --- | --- | --- | --- |
| Personnel | Staff | The proportion of medical staff is unreasonable. | 2 | 3 | 2 | 12 | Low risk |
|  |  | The structure of medical staff is unreasonable. | 3 | 3 | 2 | 18 | Low risk |
|  |  | Lack of knowledge related to hospital infection. | 6 | 6 | 4 | 144 | Medium-high risk |
|  |  | Poor aseptic awareness among medical staff. | 6 | 7 | 4 | 168 | High risk |
|  |  | Inappropriate dress code. | 6 | 3 | 2 | 36 | Medium-low risk |
|  | Patient | Contaminated or dirty surgical incisions. | 2 | 7 | 1 | 14 | Low risk |
|  |  | Many personal risk factors. | 7 | 6 | 2 | 84 | Medium risk |
| Equipment | | Failure of the air conditioning purification system or inadequate maintenance | 5 | 7 | 5 | 175 | High risk |
|  |  | Inappropriate disinfection procedures for anesthesia machines | 7 | 6 | 4 | 168 | High risk |
|  |  | Inappropriate use and handling of laryngoscopes | 3 | 4 | 3 | 36 | Medium-low risk |
|  |  | Inappropriate use of disposable instruments | 4 | 4 | 4 | 64 | Medium risk |
|  |  | Inappropriate pre-treatment of surgical instruments | 6 | 6 | 3 | 108 | Medium-high risk |
|  |  | Unqualified cleaning and sterilization of surgical instruments | 2 | 8 | 2 | 32 | Medium-low risk |
|  |  | Improper management of external instruments | 1 | 7 | 3 | 21 | Low risk |
| Material | | Inappropriate storage of sterile items | 2 | 4 | 4 | 32 | Medium-low risk |
|  |  | Improper use of sterile items | 4 | 4 | 3 | 48 | Medium risk |
|  |  | Inappropriate use of disinfectant solution | 6 | 4 | 5 | 120 | Medium-high risk |
|  |  | Unqualified handling of cleaning tools | 7 | 4 | 5 | 140 | Medium-high risk |
| Method | Management mechanism | The hospital infection management system and procedures are not complete. | 4 | 6 | 4 | 96 | Medium-high risk |
|  |  | The operating room regulations and norms are not fully implemented. | 6 | 6 | 5 | 180 | High risk |
|  |  | The management team of the department fails to fulfill their management responsibilities properly. | 6 | 5 | 5 | 150 | Medium-high risk |
|  | Surgical management | High surgical level | 2 | 4 | 6 | 48 | Medium risk |
|  |  | Large amount of intraoperative bleeding | 4 | 4 | 6 | 96 | Medium-high risk |
|  |  | Improper use of antibacterial drugs | 2 | 4 | 6 | 48 | Medium risk |
|  |  | Unstable intraoperative body temperature | 2 | 5 | 4 | 40 | Medium-low risk |
|  |  | Unsatisfactory blood sugar control during the perioperative period | 3 | 6 | 4 | 72 | Medium risk |
|  | Medical waste management | Incorrect classification and disposal of medical waste | 2 | 2 | 4 | 16 | Low risk |
|  |  | No use of dedicated channels for transferring medical waste | 2 | 2 | 4 | 16 | Low risk |
|  |  | Inappropriate handling of medical waste from infected patients | 2 | 3 | 4 | 24 | Medium-low risk |
| Environment | | The surface cleaning and disinfection in the operating room were not properly carried out. | 7 | 5 | 5 | 175 | High risk |
|  |  | The air self-purification standard was not met between consecutive surgeries. | 7 | 6 | 4 | 168 | High risk |
|  |  | The temperature and humidity in the operating room did not meet the standards. | 6 | 5 | 4 | 120 | Medium-high risk |
|  |  | The number of personnel in the operating room exceeded the regulations. | 2 | 4 | 6 | 48 | Medium risk |
|  |  | The operating room door was opened frequently during the surgery. | 6 | 2 | 6 | 72 | Medium risk |
|  |  | The arrangement of infection prevention surgeries was unreasonable. | 4 | 6 | 4 | 96 | Medium-high risk |
| Monitor | Environmental Monitoring | The environmental surface microbial monitoring did not meet the standards. | 1 | 6 | 4 | 24 | Medium-low risk |
|  |  | The air monitoring did not meet the standards. | 1 | 6 | 4 | 24 | Medium-low risk |
|  |  | The monitoring methods were incorrect. | 2 | 6 | 4 | 48 | Medium risk |
|  |  | No monitoring was conducted. | 1 | 6 | 4 | 24 | Medium-low risk |
|  | Disinfectant medical devices | The disinfectant was not monitored as required. | 8 | 5 | 4 | 160 | High risk |
|  |  | The sterilization monitoring of sterilized instruments was not standardized. | 1 | 6 | 1 | 6 | Low risk |
|  |  | The quality monitoring of surgical instrument disinfection was not standardized. | 1 | 6 | 1 | 6 | Low risk |
|  |  | The cleaning quality of surgical instruments was unqualified. | 1 | 6 | 2 | 12 | Low risk |
|  | Quality monitoring | Low hand hygiene compliance rate | 7 | 6 | 5 | 210 | High risk |
|  |  | Incorrect surgical hand disinfection | 7 | 5 | 5 | 175 | High risk |
|  |  | Inappropriate preoperative skin preparation | 6 | 5 | 5 | 150 | Medium-high risk |
|  |  | Inappropriate disinfection of the surgical area skin | 6 | 5 | 6 | 180 | High risk |
|  |  | Inappropriate aseptic operation during surgery | 6 | 5 | 4 | 120 | Medium-high risk |
|  |  | Improper use of personal protective equipment | 2 | 4 | 6 | 48 | Medium risk |
